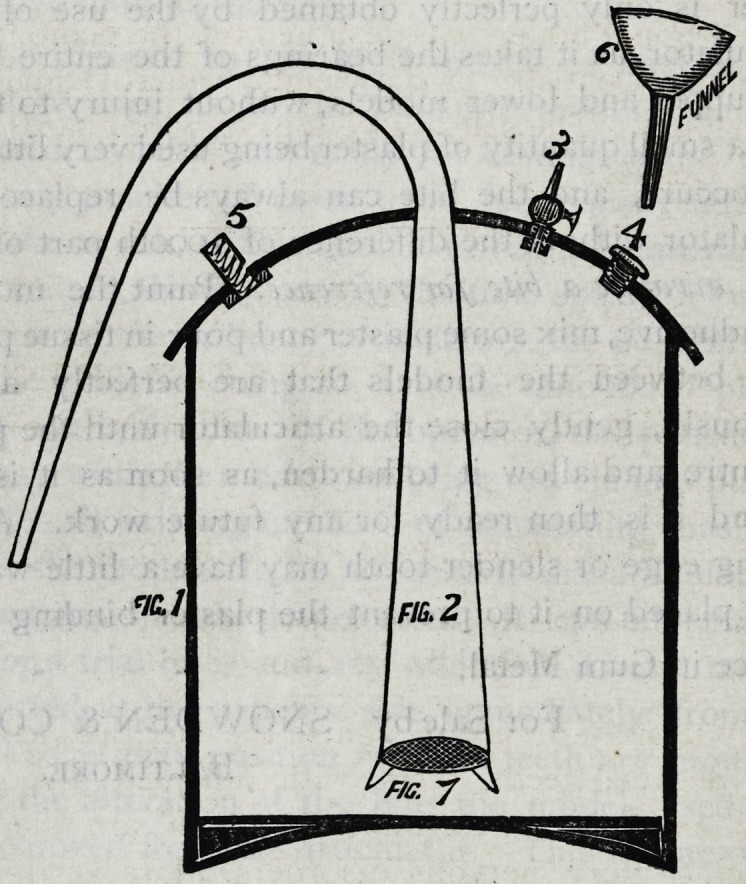# Steamer for Dental Purposes

**Published:** 1883-12

**Authors:** 


ARTICLE III.

### STEAMER FOR DENTAL PURPOSES.

THE INVENTION OF DR. D. GENESE.

FIG. 1. A copper boiler, bottom concave, top convex, soldered steam tight.

FIG. 2. A tube placed ½ an inch from the bottom passing through the top or lid, and soldered to it, curving over and ending in a blow-pipe tip, two-thirds down thc boiler, to prevent foreign matter being carried from the boiler to stop the fine hole, and circle of copper guage is fastened to the large end of the pipe marked Fig. 7.

FIG. 3. An ordinary steam tap which is left open until the water boils, and to control the flow of steam from the tube, and to empty the boiler.

No. 4. A screw nut and worm for filling the boiler.

No. 5. A safety-valve arranged by a spiral spring pressing on a disk at the lower end, and against a plate fastened to the boiler by means of the uprights, all securely soldered to the top and arranged to blow off at a temperature of 230° Fahr.

By this arrangement a stream of water is forced through the blowpipe point at a high pressure and above the boiling point, perfectly forcing out all wax, composition, or loose pieces of plaster from the flask.

The pressure is obtained by steam generating above the water, and forcing it through the large opening of the tube, it is, therefore, necessary that it should be well-constructed and strong. Copper tinned inside is the best, and all the joints should be hard soldered.

The original boiler was designed by the demonstrator of Mechanical Dentistry of the Pennsylvania Dental College, Philadelphia. The safty-valve tops (a wire guard) arranged by Dr. Genese.

## Figures and Tables

**Figure f1:**